# Halogen Bonding in Bicomponent Monolayers: Self-Assembly of a Homologous Series of Iodinated Perfluoroalkanes with Bipyridine

**DOI:** 10.1021/acs.langmuir.0c02126

**Published:** 2021-01-06

**Authors:** Jonathan A. Davidson, Marco Sacchi, Fabrice Gorrec, Stuart M. Clarke, Stephen J. Jenkins

**Affiliations:** † Department of Chemistry, University of Cambridge, Cambridge, United Kingdom; ‡ Department of Chemistry, University of Surrey, Guildford, United Kingdom; § MRC Laboratory of Molecular Biology, Cambridge, United Kingdom; ∥ BP Institute, University of Cambridge, Cambridge, United Kingdom

## Abstract

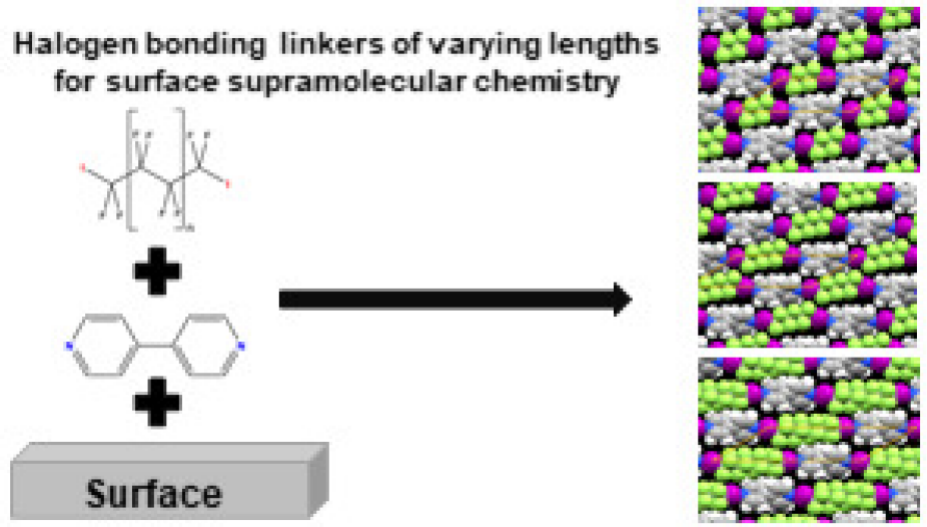

A homologous series of halogen bonding monolayers based on terminally iodinated perfluoroalkanes and 4,4′-bipyridine have been observed on a graphitic surface and noninvasively probed using powder X-ray diffraction. An excellent agreement is observed between the X-ray structures and density functional theory calculations with dispersion force corrections. Theoretical analysis of the binding energies of the structures indicate that these halogen bonds are strong (25 kJ mol^–1^), indicating that the layers are highly stable. The monolayer structures are found to be distinct from any plane of the corresponding bulk structures, with limited evidence of partitioning of hydrocarbon and perfluoro tectons. The interchain interactions are found to be slightly stronger than those in related aromatic systems, with important implications for 2D crystal engineering.

## Introduction

Self-assembly has been an area of growing interest in a variety of fields. Of particular note are 2D networks of organic molecules at surfaces, as they have potential applications in areas such as optoelectronics, catalysis, and sensing.^1^ The molecular precursors are weakly bound to the surface, limiting translational motion to two dimensions. On flat substrates with a relatively uniform surface potential the in-plane structure of these systems is governed chiefly by the adsorbate–adsorbate interactions. As these interactions are noncovalent in nature they are reversible, and thus, the observed structure is generally at thermal equilibrium.

A range of noncovalent interactions have been used to control the assembly of physisorbed molecules at surfaces. Van der Waals,^2^ dipolar^3−6^ metal coordination,^7^ and hydrogen bonding^8−10^ interactions have all been used to assemble a variety of monolayers on surfaces. The use of halogen bonding in monolayer self-assembly has been a comparatively recent development.^11^

A halogen bond is defined as an attractive interaction between the electrophilic region associated with a halogen atom in a molecule (termed the σ-hole) and a nucleophilic region of high electron density on the same or different molecule.^12^ By analogy with hydrogen bonding terminology, the electrophilic atom/molecule is termed the halogen bond donor, and the nucleophilic atom/molecule is termed the halogen bond acceptor.^13^ This interaction has enjoyed a rise in prominence in recent years, with halogen bonding identified in solid state,^14,15^ solution phase,^16^ and even biological systems.^17^

Reports of in-plane halogen bonding in monolayers have been comparatively rare and generally focused on monocomponent systems.^18^ Attempts to directly translate hydrogen-bonded assemblies to halogen bonding motifs were only partially successful,^19^ demonstrating the need to better understand the use of this interaction for 2D crystal engineering. A recent study utilized a combination of high-resolution scanning tunnelling microscopy with DFT simulation to resolve the balance between hydrogen and halogen bonding.^20^

Clarke and co-workers were the first to report a bicomponent halogen-bonded monolayer^21^ and have subsequently reported a number of additional monolayer structures formed between 4,4′-bipyridine (BPY) and a variety of aromatic halogen bond donors.^22,23^ It was observed that the strength of halogen bonding increases as one descends the group VII halogens and that electron-withdrawing groups on the halogen-containing aromatic molecule enhance the halogen bond strength.

For monolayer systems the combination of experimental diffraction studies with theoretical studies through density functional theory (DFT) is extremely powerful. X-ray diffraction provides an experimental method that obtains ensemble average values of the structure and periodicity of the monolayer. This loses some fine detail of defects and behavior at grain boundaries that scanning probe techniques provide, but it provides an ideal picture of the equilibrium structure. The invasiveness of X-rays is the source of some debate; however, for these systems we have not noticed any changes in the obtained pattern after repeated imaging. Indeed, the negligible interaction between the X-rays and matter are one of the key challenges of application of this technique to monolayer systems. Once X-ray diffraction has been used to experimentally characterize the monolayer, DFT is able to validate the modeled structure and importantly can be used to establish the contribution of different intermolecular interactions to better understand the driving forces for the assembly.

Other than scanning probe techniques and the aforementioned DFT/diffraction methodology, the two other techniques that have been used to study halogen bonding in surface systems are solid-state nuclear magnetic resonance (ssNMR) and X-ray photoelectron spectroscopy (XPS).^24,25^ Generalization of these techniques to the systems reported in this work is not straightforward. ssNMR provides a powerful tool that has been used to examine the shift of the fluorine signals in α-iodoperfluorocarbons adsorbed on silicon nitride^24^ and silica.^25^ However, the electronic effect of the graphite substrate means that for our systems the signals are broadened by significantly more than the expected shift of the fluorine signal (2–6 ppm), limiting the useful information that can be extracted. We are grateful to our colleagues (see acknowledgments) for these insights, and for preliminary experiments to confirm this effect. XPS experiments on adsorbed monolayers depend on the relative amounts of monolayer to substrate atoms near the surface for sufficient signal to be detected. Preliminary experiments on our systems proved inconclusive, indicating the challenge inherent in obtaining sufficient monolayer signal relative to the background for systems such as these.

Using a similar diffraction method to that mentioned above, the assembly of perfluoroalkane monolayers on graphite has been previously reported.^26^ Due to the large steric bulk of the fluorine atoms these chains adopt a helical structure rather than the characteristic *trans*-chain configuration of alkanes. They are observed to form close-packed solid monolayers on graphite, though exhibit a comparatively low adsorption energy, and hence are displaced from the surface by the stronger binding hydrocarbon alkanes.^27^ This low adsorption energy is symptomatic of the low polarizability of fluorine and, hence, the weak van der Waals (vdW) interactions present between the chains. This relative weakness of the intermolecular vdW interactions is key to crystal design, as it would be hoped to reduce the energetic cost in deviating from close-packing when forming a porous system. This is often a key problem encountered when designing self-assembled layers; for example, a recent study found the porous structures observed were stable only when the pores are filled with solvent or guest molecules.^28^

The assembly of halogen-bonded cocrystals between 4,4′-bipyridine and short chain α, ω-diiodinated perfluoroalkanes has been previously reported in the bulk.^29^ It was found that the chains remained in the low energy linear conformation, and segregation between the hydrocarbon and perfluorinated segments was evident.

**Figure 1:**
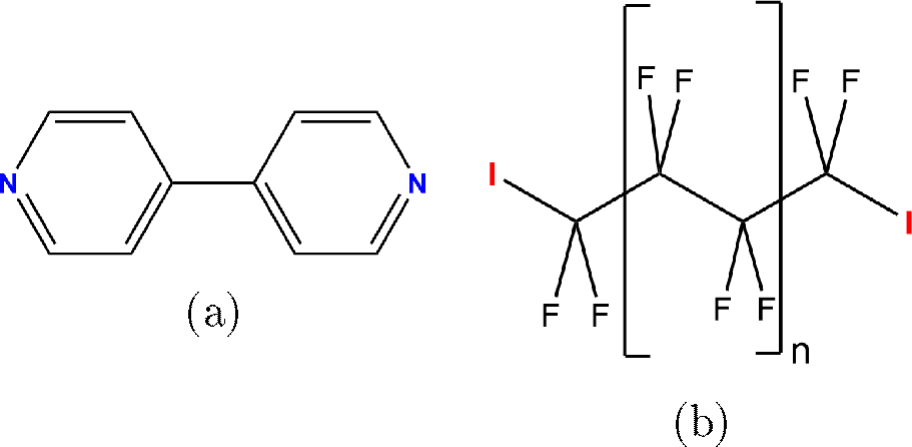
Chemical structure of (a) 4,4′-bipyridine (BPY) and (b) the family of terminally iodinated perfluoroalkanes used in this study, where *n* = 1, 2, and 3 were studied. Halogen bond donating and accepting motifs are indicated.

Here, we present a study of the monolayer assembly of a series of α, ω-terminally iodinated perfluoroalkanes (Figure 1b) with 4,4′-bipyridine (BPY) (Figure 1a) on graphite. In this work we shall designate the α, ω-diiodinated perfluoroalkanes with a name of the formula CnF2*n*I2 (e.g., C4F8I2) where *n* indicates the number of carbon atoms in the alkyl chain. All of these species are fully fluorinated, aside from the two iodine atoms.

## Experimental Section

### Diffraction

The experimental method used in this work to obtain physisorbed layers on graphite has been detailed elsewhere.^22^ The graphite substrate used was Papyex, an exfoliated recompressed graphite foil from Le Carbon. The structure of Papyex is such that the graphite crystallites are highly aligned in the plane of the sheet. This allows manipulation of diffraction geometry to maximize scattering from the in-plane monolayer peaks. The batch of Papyex used in this work was 0.5 mm in thickness and found to have a BET surface area of 15.61 m^2^ g^–1^. The adsorbates used were purchased from commercial suppliers and used without further purification. Stated purities were 98% for 4,4′-bipyridine (Alfa Aesar), 97% for C4F8I2 (Fluorochem), 97% for C6F12I2 (Alfa Aesar), and 98% for C8F16I2 (Sigma-Aldrich).

Dosing was performed through the vapor phase. Graphite and a weighed amount of the relevant adsorbates were loaded into Pyrex tubes, which were evacuated to a pressure of ca. 0.1 mbar and sealed under vacuum. The tubes were then heated to 393 K, before being left to cool slowly to room temperature to anneal. After cooling, the tubes were opened and the dosed Papyex recovered. Coverage is defined relative to complete coverage of the surface one layer thick (one ML). Components were initially weighed out such that approximately 0.8 ML coverage was achieved, using estimates of molecular areas. Calculations using the experimentally obtained lattice parameters confirm that submonolayer coverages were dosed for all systems.

In this study, a Rigaku rotating copper anode diffractometer with a graphite monochromator and MAR-DTB image-plate detector at the Laboratory of Molecular Biology (LMB) in Cambridge was used, as described previously.^30^ The sample geometry was flat plate transmission, with a nitrogen cryostream used to cool the sample to 100 K. Sample attenuation can be shown to be negligible. Calibration of the detector angles was performed using a Papyex strip coated in silver behenate.

Integration of the obtained powder rings onto a single radial dimension was performed using the fit2D software platform.^31,32^ Further analysis of the data was then performed using a custom Python script “PatternNx” that accounts for the observed “sawtooth” line shape of 2D diffraction peaks.^33^ The scattering of a bare graphite sample was subtracted from the obtained patterns. Thus, the observed peaks originate from changes due to the addition of the adsorbate.

### Computational

The periodic boundary conditions DFT code CASTEP^34^ was used to optimize the lattice parameters for the systems studied. Given the relative chemical inertness of the graphitic substrate and the flatness of the potential energy surface suggested by the experimental results, we have modeled the three self-assembled systems as rafts without explicitly considering the surface–adsorbate interaction. We used the Perdew–Burke–Ernzerhof^35^ exchange-correlation functional with a 400 eV kinetic energy cutoff. We applied the Tkatchenko Scheffler (TS) dispersion force corrections^36^ to account for long-range correlation effects (vdW interactions). These pairwise corrections are necessary due to the semilocal nature of standard GGA functionals and have proven to be robust for these types of systems.^23^

During the geometry optimization the forces are converged with a tolerance of 0.05 eV Å^–1^, with an electronic energy tolerance of 10^–5^ eV. The optimizations were left unconstrained to test the robustness of the initial structural model.

In order to estimate the contribution of different interactions to the total binding energy, the binding energy of a complete tiling can be compared to that of a system with doubled interchain spacing (*b* lattice parameter). This will (almost) eliminate the interchain interactions, and hence, the calculated binding energy will represent the strength of the two intrachain halogen bonds.

## Results and Discussion

### Monocomponent Systems

Before two component systems can be studied, it is convenient to first measure the diffraction patterns of the single component systems on graphite. This can help confirm whether or not mixing has occurred in the multicomponent system, as well as provide information on the behavior of the adsorbates as a single phase.

### 4,4′-Bipyridine

The diffraction pattern of a monolayer of BPY has been reported previously.^37^ The unit mesh was determined to be square, with lattice parameters *a* = *b* = 11.42(2) Å and γ = 90.0(2)°. The most intense peaks were those found at *Q* = 0.77 and 1.23 Å^–1^. The presence or absence of these peaks in the codeposited diffraction pattern will thus indicate the extent of mixing.

### Halogen Bond Donors

**Figure 2:**
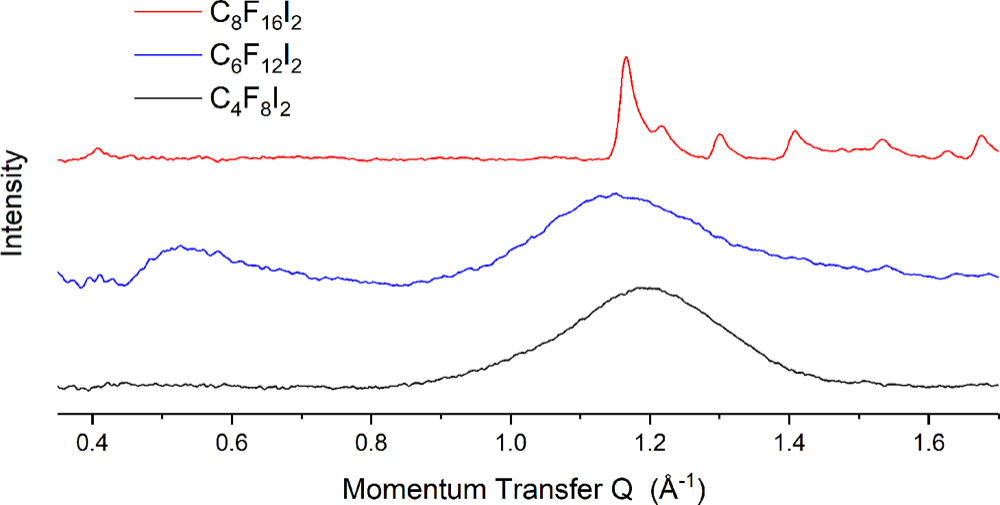
Comparison of the diffraction patterns obtained for graphite dosed with the series of halogen bond donors used in this study. Only the C8F16I2 pattern shows evidence of crystalline monolayer.

Figure 2 presents the observed patterns for graphite dosed separately with the three halogen bond donors. Incomplete subtraction of the strong 002 graphite peak at *Q* = 1.8 Å^–1^ limits the high *Q* range of the pattern. Small-angle Porod scattering is evident which limits the low *Q* range. However, no significant features were observed below *Q* = 0.3 Å^–1^.

Long chain (*n* = 6+) perfluorocarbons have been observed to lie flat on the surface of graphite.^26^ By contrast, monoiodinated perfluorinated molecules have been observed to form upright layers on silicon nitride and oxide substrates.^24,25^ In these cases, halogen bonding to the substrate was observed to lead to the chain assembling perpendicular to the surface, analogous to chemisorbed monolayers. A graphite surface does not have the same electron donating property as silica and silicon nitride; however, halogen bonding to the π systems of small molecules^38^ has been observed in bulk crystals, so an upright chain structure cannot be immediately excluded.

**Figure 3:**
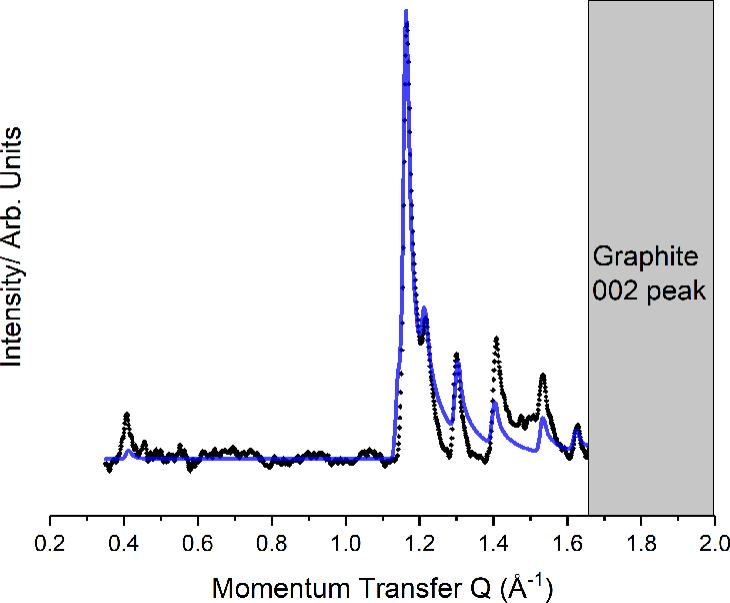
Comparison between the experimental (black) and predicted (blue) diffraction pattern for the crystalline C8F16I2 monolayer.

For the graphite dosed with C8F16I2 it is possible to discern “sawtooth” peaks in the observed pattern. The main features are at approximately *Q* = 0.4 and 1.2 Å^–1^ with a good number of weaker features. The pattern can be indexed to a unit cell with pg symmetry, with a glide plane parallel to the a lattice parameter. This unit cell has lattice parameters *a* = 31.38(2) Å, *b* = 5.52(5) Å, and γ = 90°. This is similar but slightly longer than the high symmetry Phase I centered structure previously reported for perfluorooctane.^26^ The peak intensities are well fit by a flat-lying structure with chains lying at an angle of approximately 7° to the *a* lattice parameter. Figure 3 compares the modeled intensities (black) with the experimental data (gray). Any structure incorporating perpendicular chains could not match the observed peak intensities. At room temperature this pattern was observed to melt to an amorphous pattern.

For both C4F8I2 and C6F12I2, no sharp peaks were detected, even after extended time to equilibrate in the 100 K cryostream. Broad amorphous features are observed at approximately *Q* = 1.2 Å^–1^ and 1.1 Å^–1^ respectively, with a secondary feature for C6F12I2 observed at *Q* = 0.5 Å^–1^. This is initially surprising, as the bulk melting temperatures of these compounds is −3° and 29°, respectively,^39^ and the monolayer melting temperature would be expected to be similar.^40^

To confirm the sample’s purity, differential scanning calorimetry (DSC) was performed on the purchased samples which matched the literature bulk melting temperatures. However, a large hysteresis in melting and freezing temperatures was observed. For example, C4F8I2 melted at −3° yet only froze at −28° in the DSC. This thermal hysteresis is indicative of difficulty crystallizing in the bulk and indicates that kinetic trapping could also be problematic in monolayer crystallization. Given the lack of crystallization, an unambiguous structural assignment for these layers is not possible.

Overall, there is minimal evidence of crystalline monolayer formation under the experimental conditions for the C4F8I2 and C6F12I2 molecules. The larger C8F16I2 system forms a crystalline monolayer on graphite that can be identified at low temperatures.

### Cocrystals

**Figure 4:**
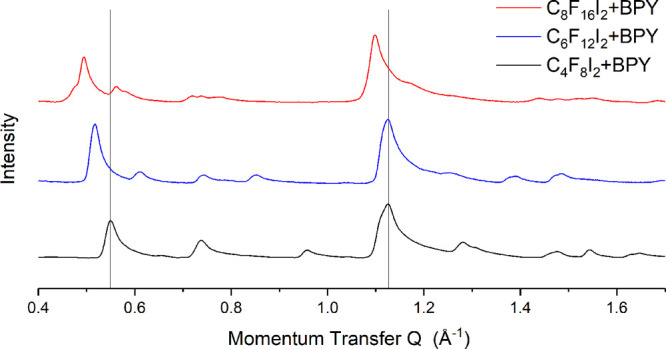
Comparison of the diffraction patterns obtained for graphite dosed with BPY alongside the series of halogen bond donors used in this study. The first peak (indexed as 01) shows a progression to lower *Q* with increasing chain length.

Figure 4 presents the diffraction pattern observed for 1:1 ratios of the C4F8I2:BPY, C6F12I2:BPY, and C8F16I2:BPY systems. Structural assignment will be performed in subsequent sections, but from initial observation it is clear that these systems exhibit diffraction patterns that are distinct from those of the individual components. This indicates a new phase is being formed. The observed peaks all exhibit the characteristic “sawtooth” shape characteristic of monolayer diffraction peaks. The distinctive shape of these peaks is diagnostic of 2D layers because the long trailing edge is associated with Bragg rods in the plane perpendicular direction. This indicates a lack of periodicity perpendicular to the plane and hence rules out any 3D structures. For each system, if an excess of one component was added, the collected pattern was a superposition of the bicomponent diffractogram from Figure 4 and the excess monocomponent phase. It is thus clear that the mixed phase contains each component in an approximately 1:1 ratio. This is in agreement with the expectations if the halogen bond is key to cocrystal formation.

By way of contrast, the brominated analogue C8F16Br2 did not show evidence of mixing with BPY, the collected diffractogram being simply an addition of the monocomponent patterns of C8F16Br2 and BPY (see Figure S1).

Additional room-temperature XRD also indicates that the structures formed are stable under ambient conditions, above the melting point of several of the individual components. This is typical of strongly bound cocrystals. These layers can therefore be considered robust. Washing with water and dodecanol was observed to only minimally disrupt the diffraction pattern of the C4F8I2:BPY system. Ethanol, a good solvent for both components, was observed to readily remove the layers. The resilience of these layers will be explored in subsequent work.

It is evident that there are two major peaks in the diffraction patterns of all three cocrystals, indicated by the two lines in Figure 4. Observing the progression of peaks, it is evident that the first peak shifts to lower *Q* with increasing length of halogen bond donor. Assuming the structures are homologous, this suggests the molecules may be partially aligned in this lattice direction.

### Data Fitting

The process of analyzing monolayer diffraction patterns has been described elsewhere.^6^ In brief, the unit mesh of the overlayer must first be indexed from the experimental peak positions. High symmetry unit cells are generally preferred, but for large unit cells this can be unwieldy for simulation, hence the use of primitive unit cells in this fitting.

The intensities of the observed peaks can then be used to populate the indexed unit mesh with the molecular adsorbates. Due to the limited number of reflections visible and the projection of the diffracted beams onto a single axis, it is necessary to constrain the fitting. The molecular structures are held to be fixed and can be treated as rigid bodies in the fitting. The structures employed are based on the relevant bulk diffraction pattern from the Cambridge Structural Database^41^ and, in particular, the 3D single-crystal structures reported by Catalano et al.^29^

In keeping with previous work, and to limit the number of degrees of freedom of the model, the Debye–Waller factors have been set to unity. Qualitatively, this term would be expected to suppress the intensity of higher *Q* peaks, and so, our fitting will slightly underestimate the intensity of low *Q* peaks. The peak shapes were modeled using the Gaussian peakshape models of Schildberg and Lauter.^33^

### C4F8I2+BPY

**Figure 5:**
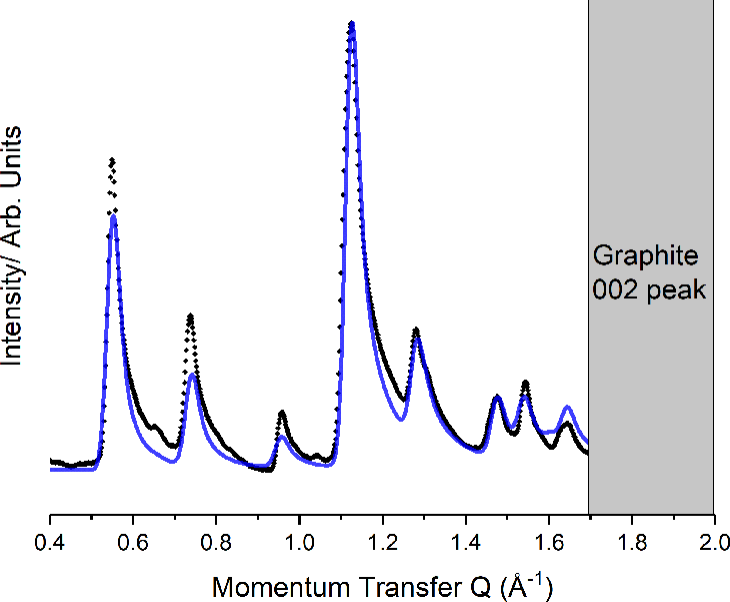
Comparison between the experimental (black) and predicted (blue) diffraction pattern for the C4F8I2:BPY cocrystal.

Figure 5 presents the observed diffraction pattern for a sample dosed with C4F8I2 and BPY. It is possible to index the pattern to an oblique unit cell with lattice parameters *a* = 20.47(8) Å, *b* = 9.99(0) Å, and γ = 34.20°. This is incommensurate with the underlying hexagonal graphite lattice *a* = 2.4589 Å and γ = 60°, indicating that intermolecular interactions are more important than the substrate in determining monolayer structure. The area of this cell matches well with the expected size of a 1:1 C4F8I2:BPY complex. Similarities can be identified between this unit cell and that previously observed for the cocrystalline monolayer formed from a mixture of 1,4-diiodotetrafluorobenzene and BPY.^21^

If one assumes that the halogen bond is important in structural determination, then a logical trial structure is one based around linear chains. It is found that the pattern is well fit by such a structure consisting of the I atoms of the C4F8I2 molecule and two N atoms of BPY lying along the same axis. Deviation from this linearity by only a few degrees dramatically worsened the fit, heavily supporting a linear structure. Interestingly, this structure showed no evidence of the segregation between hydrocarbon and perfluorinated tectons that is observed in the bulk.^29^ It has previously been noted that mixing of dissimilar components occurs more readily in 2D than in 3D, as a function of the decreased dimensionality of the system.^42^

The underestimation of low-*Q* peak intensities in this model likely indicates significant Debye–Waller factors (previously observed to be insignificant in our studies of aromatic systems). This is as expected, as less constrained fluorocarbon chains may be less rigid than an aromatic ring and so exhibit a greater degree of thermal motion.

### C6F12I2+BPY

**Figure 6:**
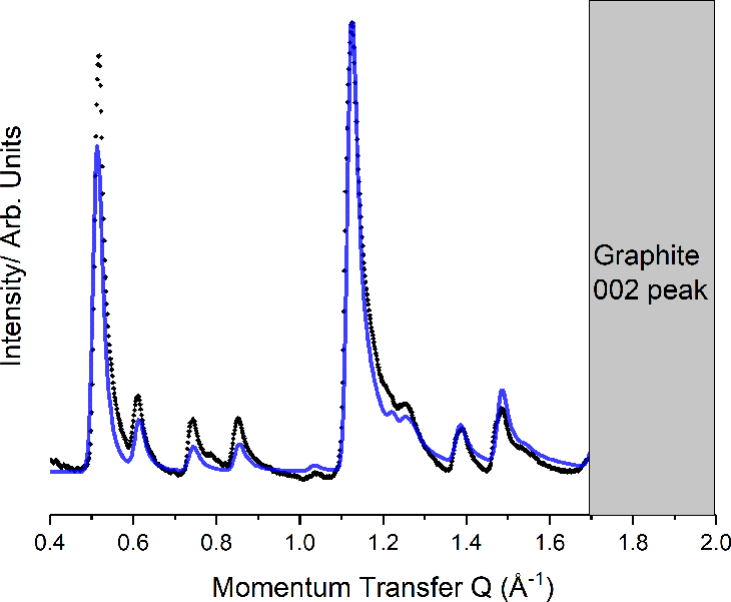
Comparison between the experimental (black) and predicted (blue) diffraction pattern for the C6F12I2:BPY cocrystal.

Similar analysis has been performed for the C6F12I2:BPY cocrystal and is presented in Figure 6. Unit mesh parameters were optimized to be *a* = 22.71(5) Å, *b* = 10.44(3) Å, and γ = 32.53°. Again, this indicates an incommensurate unit cell, implying strong intermolecular interactions. Similar to the above, it seems the low-angle peak intensities are under-predicted in our model, likely due to Debye–Waller factors. Again, any deviation from a linear chain structure dramatically worsened the fit.

### C8F16I2+BPY

**Figure 7:**
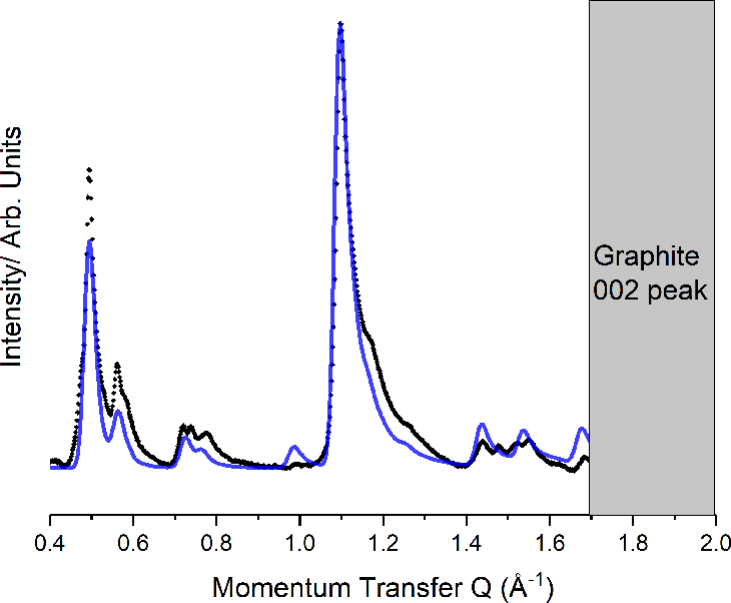
Comparison between the experimental (black) and predicted (blue) diffraction pattern for the C8F16I2:BPY cocrystal.

Figure 7 presents a similar plot for the C8F16I2 + BPY system. A fit has been performed in an analogous method to that used above to index the pattern to an incommensurate cell with parameters a = 25.27(4) Å, b = 11.29(3) Å and γ = 30.61°.

Interestingly, in this pattern the initial model (which was based on the previously published all-*trans* chain bulk C8F16I2 structure) was a poor match for the experimental data. A twisted helical chain structure has been observed in bulk by Metrangolo et al.^43^ and was able to achieve a closer match to the experimental data. Using similar twisted chain structures in the shorter systems had negligible impact on the fit, and hence, the all-*trans* chain structures were used so as to minimize fitting parameters.

Incidentally, a search of bulk perfluoroalkyl chains in the CSD indicates that a surprisingly large proportion of reported structures feature perfluoroalkyl chains with a torsion angle of exactly 180°, indicating perhaps the difficulties of fitting exact torsion angles of perfluoroalkyl chains using diffraction, even for bulk single crystals. In these cocrystals, the halogen-bonded interactions are usually of most interest.

### Summary of Diffraction Data

C8F16I2 forms a crystalline monolayer, but this was only observed at low temperature. However, interestingly, neither C4F8I2 or C6F12I2 showed evidence of crystalline monolayer formation in the absence of a halogen bond acceptor.

When codeposited with the halogen bond acceptor BPY, all three molecules showed clear evidence of new monolayer cocrystalline phases. For the three systems studied, the diffraction patterns could be indexed to a similar set of unit meshes. Peak intensities were fit to a set of homologous structures containing linear chains. These meshes were significantly different to any planes of the bulk crystal structures, indicating that the monolayer structures are novel and not simply a plane of the corresponding bulk cocrystals.

The damping of higher *Q* intensities of the experimental patterns compared to the modeled structures may indicate that the structures are slightly less rigid than the previously studied aromatic systems. For the longest chain halogen bond donor, the helical nature of the perfluorinated chain had a significant effect on the observed pattern, but it was more difficult to fit a torsion angle to the shorter chain molecules.

### DFT Calculations

DFT geometry optimizations were performed using the experimental model unit cell and atomic positions as the starting point. Relaxation of the structure leads to minor changes (<1.5%) in the unit mesh lattice parameters. Remarkably little structural change was seen in the geometry-optimized structure relative to the initial model. This provides further evidence that the experimentally determined structures are reasonable.

**Figure 8:**
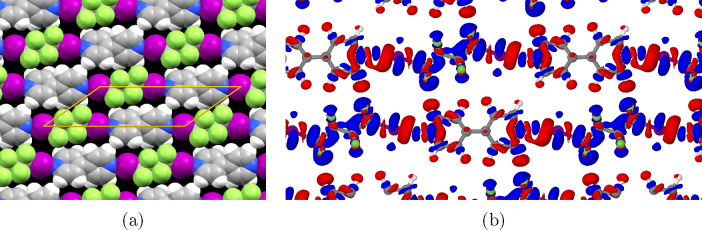
Optimized structure of the C4F8I2+BPY system. (a) The structure visualized using spacefill atomic models. (b) Plot of the electron density difference of the monolayer relative to the separate molecules. Blue indicates an increase in electron density while red indicates a loss of electron density relative to the separate molecules (isosurface level 0.005 e/Å^3^). Shifts in electron density due to the halogen bond are clearly visible, with there mainly being an increase in iodine and decrease in nitrogen electron density.

Figure 8 shows a visualization of the simulated structure of the C4F8I2:BPY layer. As in the diffraction optimized structure, the mixing of the perfluorinated and hydrocarbon tectons is evident.

By comparing the total energy of the calculated structure with that of the individual component molecules, the total binding energy of the structure can be found. Here, it is calculated that the total intermolecular interactions equate to 1.120 eV (115.7 kJ mol ^–1^) per unit cell. It is possible to estimate the interchain and intrachain components by doubling the *b* parameter to effectively remove interchain interactions, leaving only the halogen bonds (two per cell) intact. Each halogen bond can then be estimated as having a strength of 0.255 eV (24.6 kJ mol^–1^), which is similar to that previously reported for an aromatic halogen bond donor (0.249 eV^22^). The lateral hydrogen bond and vdW interactions can then be estimated as having a total strength of 0.627 eV. This is slightly larger than the previously simulated interaction for the aromatic halogen bond donor 1,4-diiodotetrafluorobenzene. In 3D crystal engineering perfluorinated aromatic molecules are considered to be more strongly interacting than their perfluoroalkane equivalents. In two dimensions it is evident that this ordering is reversed, as the exposed edge of a perfluorinated aromatic system consists of fewer, less polarizable, sp_2_ carbon bonded fluorine atoms than the perfluoroalkyl chains considered in this work.

Within the plane of the layer, the only relevant interaction with the fluorine is weak C–H bonds to the aromatic protons of BPY. Although generally considered inert, fluorine is capable of acting as a hydrogen bond acceptor, with sp^3^ C–F motifs being better acceptors than sp^2^.^44^ Such bonds are rare however, and due to their weakness they generally only occur where no other interaction is possible.^45^

The electron density difference between the bound and free molecules is a useful proxy for the nature of interactions. The plot in Figure 8b shows the difference in electron density in the cocrystal relative to the component molecules. Blue indicates an increase in electron density and red a decrease. Significant charge transfer is characteristic of halogen and hydrogen bonds, while minimal electron density difference indicates that dispersion forces are the chief method of binding. As observed previously, the halogen bond is evidenced by the increased electron density on the iodine and reduced density on the nitrogen. Hydrogen bonds are also evident in the lateral interactions, with particularly large degree of charge transfer evident between the α and β fluorine atoms and their adjacent BPY protons.

**Figure 9:**
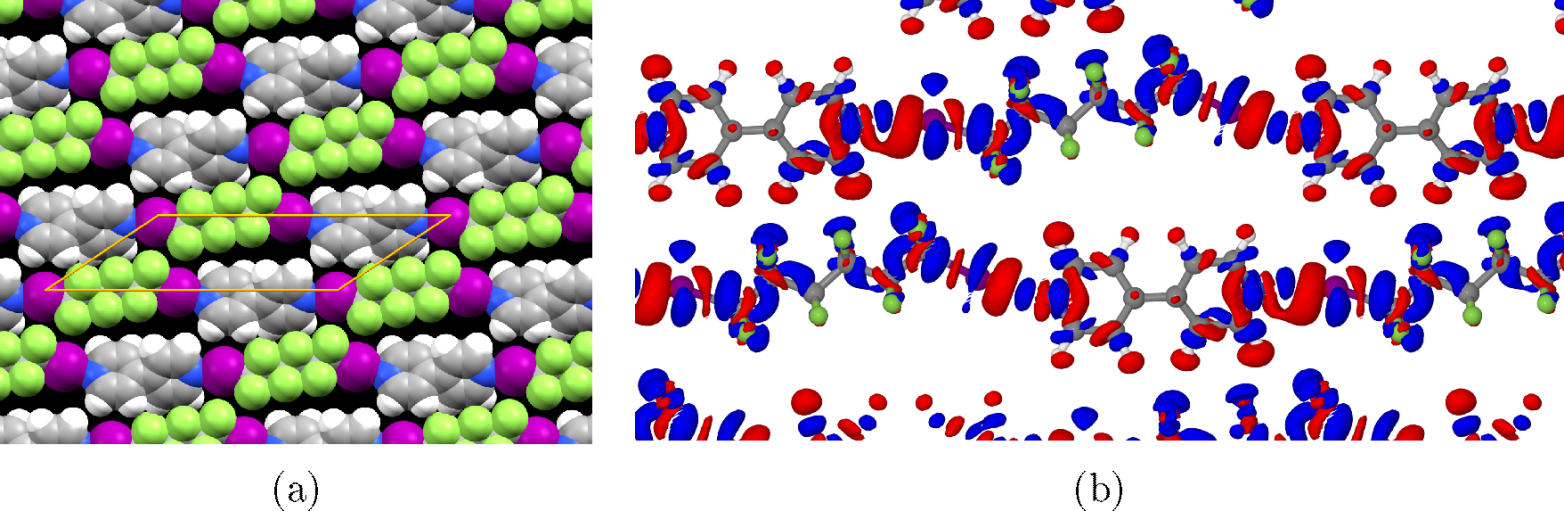
Optimized structure of the C6F12I2+BPY system. (a) The structure visualized using spacefill atomic models. (b) Plot of the electron density difference of the monolayer relative to the separate molecules. Blue indicates an increase in electron density while red indicates a loss of electron density relative to the separate molecules (isosurface level 0.005 e/Å^3^). Charge transfer between the chains is evidence of interchain hydrogen bonding between C–H and F atoms.

Similar optimization was performed for the C6F12I2+BPY system (Figure 9). Again, very good agreement is found with the experimental structures where the simulated unit cells are within 1.5% of the experimental lattice, and minimal change in molecular geometry is observed. The electron density difference plot shows a similar pattern to that observed in the shorter C4 system.

For the C8F16I2+BPY system, optimization was performed on both an all-*trans* alkyl chain analogous with the previous systems and a twisted-chain structure more consistent with the experimental data. When considering the C8F16I2 molecule individually, the twisted-chain conformer is 0.0488 eV (4.7 kJ mol ^–1^) more stable than the *trans* conformation. However, for the cocrystal the twisted conformer is 0.128 eV more stable. This means that the twisted-chain conformer exhibits a stronger binding energy to BPY than the *trans* conformer by 0.0790 eV (7.6 kJ mol ^–1^).

**Figure 10:**
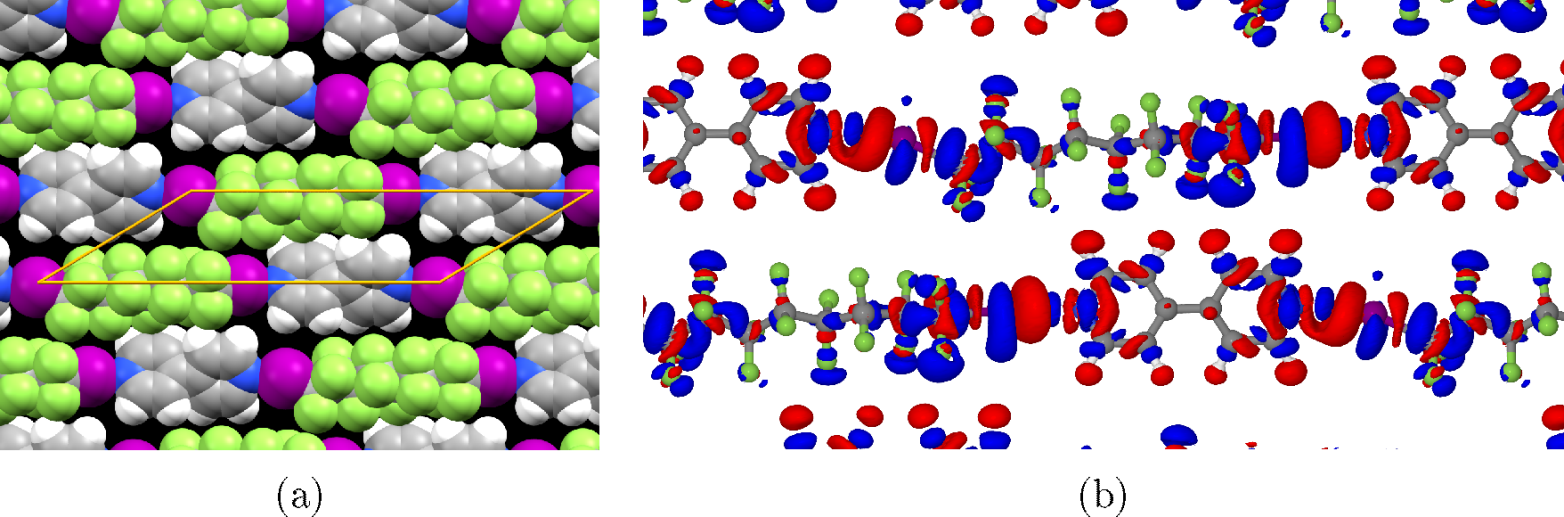
Optimized structure of the C8F16I2+BPY system. (a) The structure visualized using spacefill atomic models. (b) Plot of the electron density difference of the monolayer relative to the separate molecules. Blue indicates an increase in electron density while red indicates a loss of electron density relative to the separate molecules (isosurface level 0.005 e/Å^3^). Aside from one putative hydrogen bond, the central fluorines are relatively uninvolved with charge transfer, explaining the small energy differences between conformers.

The optimized unit cell parameters obtained for the two conformers are extremely similar, with *a* being 0.5% and *b* 0.8% larger for the *trans* conformer. As the twisted conformer is lower in energy and was the configuration that best matched the experimental data, it is the structure presented in Figure 10. The twisting of the chain largely affects the position of the fluorine atoms in the middle of the chain; however, as can be seen in the electron density difference (Figure 10b), these atoms are relatively uninvolved in charge transfer, with only one δ fluorine exhibiting a charge increase consistent with hydrogen bonding. This helps explain the comparatively small binding energy difference.

Table 1 summarizes the simulated geometry of the three unit cells, as well as the calculated binding energies. For comparison, the previously calculated data for 1,4-diiodotetrafluorobenzene (DITFB) are included. It can be seen that the halogen bonds in all three systems reported here are of similar strength to that previously calculated for DITFB. The two halogen bonds together account for almost half of the total binding energy in each case and thus make a significant contribution to the mixing of these otherwise dissimilar components.

**Table 1:** Results of the DFT-Optimized Unit Meshes for the Cocrystals Studied, Alongside Binding Energy and Estimated Halogen Bond and Interchain Interaction Strengths^a^

species	a /Å	b/Å	γ	total B.E. /eV	X-bond B.E./*eV*	interchain B.E./*eV*
C4F8I2 + BPY	20.29	9.89	34.99	1.199	0.255	0.627
C6F12I2 + BPY	22.81	10.54	33.51	1.168	0.256	0.658
C8F16I2 + BPY	25.46	11.34	30.96	1.130	0.253	0.624
DITFB + BPY	19.36	12.45	31.73	1.078	0.249	0.580

a For each system the total binding energy consists of two halogen bonds plus the total interchain interaction energy. Data previously reported for 1,4-diiodotetrafluorobenzene (DITFB) have been reproduced for comparison.^22^

The total binding energies for the three systems are remarkably similar. A doubling of the carbon chain length between C4F8I2:BPY and C8F16I2:BPY leads to a slight *reduction* in interchain interaction. This indicates that the vast bulk of the interchain interactions originate from hydrogen bonds to the BPY, with little contribution to the binding from non-hydrogen bonding atoms. This has important implications for future rational design of monolayers containing perfluorinated motifs.

## Conclusion

Using a combination of simulation and experimental techniques, we have characterized the assembly of a series of bicomponent halogen-bonded monolayers on a graphite surface. The cocrystalline monolayers with bipyridine are more robust than the monolayers of the halogen bond donors alone, maintaining their crystallinity above the bulk melting point. Compared to the corresponding bulk cocrystals, the monolayers exhibit novel packing with a greater degree of mixing between dissimilar components.

These experiments help demonstrate that crystalline monolayers can be formed using nonaromatic halogen-bond-donating groups, providing a new category of molecules that can be used in 2D crystal engineering. As supramolecular linkers, these molecules are more customizable in terms of length than their aromatic counterparts, as well as possessing different electronic and other properties that may be useful in future applications of halogen bonding in surface supramolecular chemistry. However, the comparatively high interchain interaction energies indicate that these linkers may not represent as significant a step toward the formation of porous layers as initially hoped.

## Supporting Information Available

(Figure S1) Diffractogram of the C8F16Br2+BPY system (PDF)
