# Supplementary material for: Halogen Bonding in Bicomponent Monolayers: Self-Assembly of a Homologous Series of Iodinated Perfluoroalkanes with Bipyridine
Source: Langmuir. 2021 Jan 6;37(2):627–35. doi: 10.1021/acs.langmuir.0c02126 (PMC8397337; doi:10.1021/acs.langmuir.0c02126)

Supporting Information

Halogen Bonding in Bicomponent Monolayers: Self-Assembly of a Homologous Series of Iodinated Perfluoroalkanes with Bipyridine

Jonathan A. Davidson[a,*], Marco Sacchi[b], Fabrice Gorrec[c], Stuart M. Clarke[a,d], and Stephen J. Jenkins[a]

[a] *Department of Chemistry, University of Cambridge, Cambridge, United Kingdom*

[b] *Department of Chemistry, University of Surrey, Guildford, United Kingdom*

[c] *MRC Laboratory of Molecular Biology, Cambridge, United Kingdom*

[d] *BP Institute, University of Cambridge, Cambridge, United Kingdom*

*Corresponding author email: **jad81@cam.ac.uk**

Figure-S1 Diffractogram of graphite dosed with C8F16Br2 + BPY showing phase separation, indicated by the presence of the relevant monocomponent patterns and absence of any new peaks in the co-deposited diffractogram.

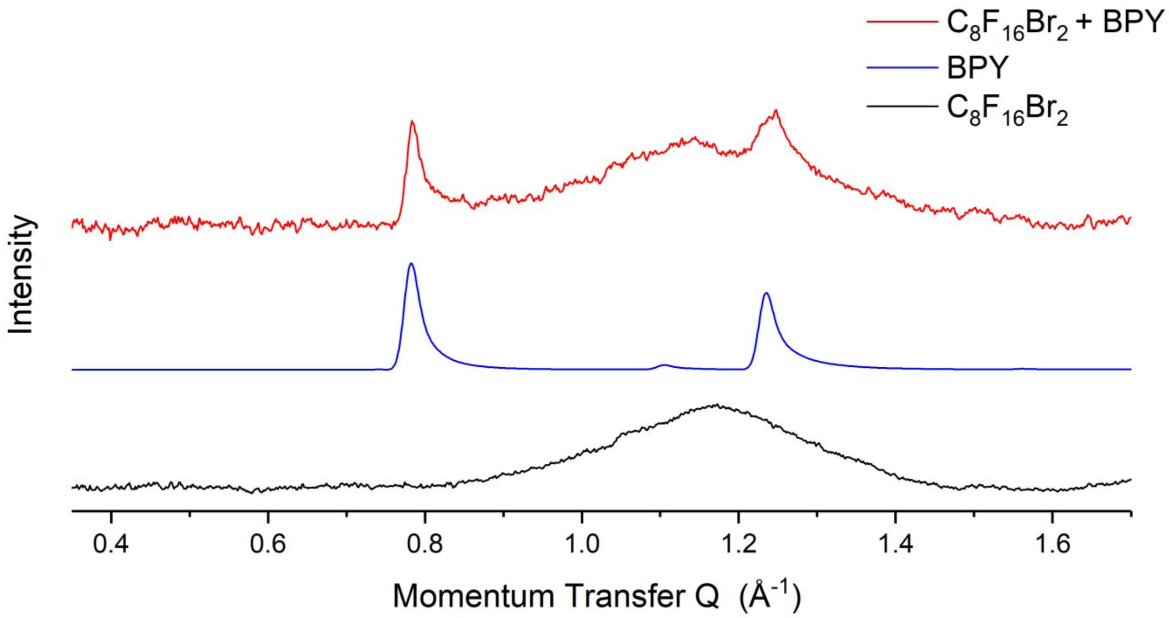

Supplement: Supplementary file 1 — la0c02126_si_001.pdf [file la0c02126_si_001.pdf]
